# MicroRNA‐181a/b‐1‐encapsulated PEG/PLGA nanofibrous scaffold promotes osteogenesis of human mesenchymal stem cells

**DOI:** 10.1111/jcmm.16595

**Published:** 2021-05-14

**Authors:** Peiyi Qi, Yali Niu, Bin Wang

**Affiliations:** ^1^ Department of Emergency Surgery the First Affiliated Hospital of Zhengzhou University Zhengzhou China; ^2^ Department of Lung Transplantation Surgery the First Affiliated Hospital of Zhengzhou University Zhengzhou China; ^3^ Department of Thoracic Heart Surgery Changyi People's Hospital Weifang China

**Keywords:** bone, electrospinning, microRNAs, nanofibres, PLGA, tissue engineering

## Abstract

Bioactive nanofibres play a useful role in increasing the efficiency of tissue engineering scaffolds. MicroRNAs (miRs) alone, and in combination with tissue engineering scaffolds, can be effective in treating bone fractures and osteoporosis by regulating many post‐transcriptional cellular pathways. Herein, miR‐181a/b‐1 was incorporated in the electrospun poly (lactic‐co‐glycolic acid) (PLGA) nanofibres (PLGA‐miR). After characterization scaffolds, the osteoinductive capacity of the nanofibres was investigated when adipose‐derived mesenchymal stem cells (AT‐MSCs) were cultured on the PLGA and PLGA‐miR nanofibres. miR incorporating in the nanofibres has not any significant effect on the size and morphology of the nanofibres, but its biocompatibility was increased significantly compared to the empty nanofibres. Alkaline phosphatase (ALP) activity and calcium measures were evaluated as two important osteogenic markers, and the results revealed that the highest measures were observed in the AT‐MSCs cultured on PLGA‐miR nanofibres. Detected ALP activity and calcium measures in miR‐transduced AT‐MSCs cultured on TCPS were also significantly higher than AT‐MSCs cultured on PLGA and TCPS groups. The highest expression levels of bone‐related genes were observed in the AT‐MSCs cultured on PLGA‐miR nanofibres. This improvement in the osteogenic differentiation potential of the AT‐MSCs was also confirmed by evaluating osteopontin protein in the cells cultured on PLGA‐miR. It can be concluded that miR‐181a/b‐1 has a significant impact on the AT‐MSC osteogenic differentiation, and this impact synergistically increased when incorporated in the PLGA nanofibres.

## 1 INTRODUCTION

Due to the growing trend of bone lesions and the low efficiency and limitations of existing treatment methods, tissue engineering in recent decades has tried to play an effective role in this field.^1^ Tissue engineering, in addition to providing the substrates that facilitate stem cell differentiation by producing scaffolds that are compatible with target tissues, can also provide this process more efficient with scaffolds that carry differentiating factors and release them in situ.^2^, ^3^ Delivery with nanofibre carriers is one of the new in situ drug delivery techniques.^4^ Various factors can be carried by electrospun nanofibres, including chemical and genetic factors as one of the most important components of tissue engineering. Among the genetic factors, microRNAs (miRs) have attracted growing attention in recent years, due to the beneficial effects they can have in the process of tissue repair.^5^ Nanofibres can structurally help differentiation of stem cells by creating an appropriate microenvironment and by inducing signalling pathways such as Wnt/β‐catenin and bone morphogenic protein 2 (BMP2). Nanofibres can also facilitate the adhesion, proliferation and differentiation of stem cells.

miRs are 19‐24 nucleotides long, single‐stranded non‐coding RNA molecules that regulate the expression of target genes by binding to 3′UTRs and possibly 5′UTR mRNAs.^6^ In humans, about 10%‐30% of the genome is controlled by miRs.^7^ The miRs show different expression patterns in various tissues and are involved in vital cellular processes such as proliferation, cellular ageing, apoptosis, metabolism and cell differentiation.^8^, ^9^ Several signalling mediators that regulate osteogenesis, including Notch ligands, bone morphogenic protein (BMP), Wnt ligands, hormones and growth factors such as tumour necrosis factor (TNF), transforming growth factor (TGF) and cytokines, can be induced by various microRNAs. Findings from numerous studies on the role of miRs in the process of bone differentiation of mesenchymal stem cells (MSCs) have shown that some of them such as miR‐30b and c, miR‐34c, miR‐205, miR‐217, miR‐137‐3p, miR‐9‐5p and miR‐23a cause a decrease and/or suppression of differentiation efficiency in the MSCs.^10^, ^11^, ^12^, ^13^ Some others, such as miR‐148b‐3p, miR‐1297, miR‐21‐5p, miR‐129‐5p, miR‐378‐5p, miR‐2861, miR‐22 and miR‐497‐5p, cause an increase degree of osteogenic differentiation in stem cells.^14^, ^15^, ^16^, ^17^, ^18^, ^19^

One of the most difficult bone lesions to treat is a fracture or lesion that develops in long bones. Of note, a high expression of miR‐181 has been found during the development of long bones, and thus, it is predicted to play a role in repairing bone lesions.^20^ In this study, miR‐181a/b‐1 was encapsulated in electrospun poly (lactic‐co‐glycolic acid) (PLGA) nanofibres. PLGA is a biodegradable polyester that is approved by the US Food and Drug Administration (FDA) and the European Medical Agency (EMA) for use in various human drug delivery systems.^21^ This copolymer is composed of two polymers, polylactic acid and polyglycolic acid, which are also used as scaffolds in tissue engineering and in the construction of nerve conduction channels alone or in combination with other materials. PLGA is highly biocompatible and biodegradable, while its degradability time can be controlled from a few weeks to several years based on the ratio of polylactic acid to polyglycolic acid.^22^, ^23^ This substance is slowly hydrolysed in the body, degraded and metabolized to lactic acid and glycolic acid monomers, and finally excreted as carbon dioxide and water through the Krebs cycle.^24^ The aim of the present study was to fabricate and characterize the PLGA and PLGA‐miR nanofibrous scaffolds as a promising bio‐implant for bone tissue engineering. To this end, the osteogenic differentiation of the adipose‐derived MSCs (AT‐MSCs) cultured on the fabricated scaffolds was evaluated by measuring the common osteogenic markers.

## 2 MATERIALS AND METHODS

### 2.1 Electrospinning

Electrospinning^25^ was applied to fabricate bilayer PLGA‐gelatin/miR‐181a/b‐1 nanofibrous scaffolds. The outer layer was PLGA, and the inner layer was the polyplex/miR/gelatin. To prepare the outer layer, PLGA (MW 50 000, 50:50 monomer ratio and 0.55‐0.75 dl/g inherent viscosity; Daigang, China) at a w/v ratio of 15% was dissolved in a mixed solution of dimethylformamide /tetrahydrofuran (DMF/THF; 4/1 v/v). To prepare the polymer/miR‐polyplex, miR‐181a/b‐1 was mixed with spermidine (0.3 mg/ml) in Tris‐EDTA buffer (10 mM Tris‐HCl, 1 mM EDTA; pH 7.4) at an 8:1 polyamine nitrogen to nucleotide phosphate (N:P) ratio. To prepare the inner layer, the miR‐polyplex solution was added dropwise to the gelatin solution dissolved in TFE (10% w/v). The miR‐polyplex/gelatin solution was finally mixed with the prepared PLGA solution, wherein the final concentration of miR was 100 ng/ml. The prepared emulsion was gently vortexed before electrospinning for 15 minutes to provide a homogeneous distribution of the miR complex before electrospinning. The emulsion solutions were loaded into 5‐ml syringes and then electrospun at a voltage of 15‐18 kV, a flow rate of 0.5 ml/h and a distance of 15 cm between the collector and needle. Each layer was electrospun for 8 hours. PLGA solution without the miR‐polyplex/gelatin was also prepared and electrospun as a control.

### 2.2 Morphological characterization

The scanning electron microscopy (SEM) was used to evaluate the morphology of the fabricated scaffolds before and after cell seeding. In brief, fabricated scaffolds were cut and then covered with a thin layer of gold before placing under the microscope. Cell‐seeded scaffolds were also treated with glutaraldehyde (2.5%) for fixation and then dehydrated with an ethanol serial dilution (50°‐100°). After that, dried scaffolds were coated with a thin layer of gold and then placed under the SEM (SEM, KYKY, EM3200) for observation.

### 2.3 The release profile of miRs

Three pieces of the fabricated PLGA‐miR nanofibrous scaffold with certain weights (20 mg) were shaken and incubated in the phosphate buffered saline (PBS, 5 ml) at 37°C. On the certain time‐points, 0.5 ml of the PBS solution was removed and miR concentration was measured using RiboGreen^®^ kit according to the manufacture's protocol. It should be noted that the removed solution was replaced with the same volume of fresh PBS. To calculate the cumulative release profile of the miR, the following equation was used: Cumulative release = (m_1_ − m_2_) / m_1_ × 100%, where m_1_ and m_2_ involve a primary mass of the scaffold and the mass of scaffold on certain time‐points, respectively.

### 2.4 Protein adsorption

To protein adsorption evaluation of the fabricated scaffolds (PLGA and PLGA‐miR), scaffolds were cut into certain dimensions and then immersed in PBS and FBS, l% v/v solution for 1 h, while its initial protein content was measured at 530 nm by a microplate reader. After that, fabricated scaffolds were removed from the solution and then transferred to the new solution containing PBS and SDS for 20 hours to release the adsorbed proteins. The total protein concentration of the last solution was also read at 530 nm after 20 hours using a microplate reader. These acquired optical densities were converted to μg/mm^3^ of the scaffolds by comparing with the optical densities obtained by serial dilution of BSA/SDS/PBS solution at 530 nm.

### 2.5 Stem cell isolation and characterization

MSCs from the fat tissues of five healthy human adult volunteers (32 ± 4 years) were isolated and purified by density gradient centrifugation combined with an attachment culture method as previously explained according to the previously reported protocol.^26^ All experimental procedures were carried out based on the guidelines approved by the Ethics Committee of the First Affiliated Hospital of Zhengzhou University, Zhengzhou, Henan, China, and written informed consent was provided from all volunteers.

In detail, the isolated fat tissues were stored in the PBS solution supplemented with antibiotics and antifungal and transferred to the laboratory with a temperature of 2‐8°C. Under the laminar hood, the tissues were washed several times by fresh PBS/antibiotics followed by centrifuging to eliminate blood clots. Tissues were then digested enzymatically with collagenase type‐I solution (0.2%, Sigma‐Aldrich) while placing on the shaker for 45 minutes at 37°C. The digested tissues were centrifuged at 1600 RPM for 20 minutes at 25°C. The supernatant was removed, and the cell pellet was mixed by basal medium (DMEM and 15% FBS) and then aliquoted in the several cell culture flasks and incubated at 37°C with 5% CO_2_. Basal medium was exchanged every three days to eliminate non‐adherent cells. To characterize the isolated stem cells, they were cultured under the adipogenic and osteogenic differentiation media and after two weeks stained with Oil Red and Alizarin Red dyes, respectively, according to the previously reported protocol.^27^ The adipogenic medium consisted of DMEM containing 10% FBS, 0.5 mM hydrocortisone, 60 mM indomethacin and 0.5 mM IBMX, and the osteogenic medium was DMEM containing 10% FBS, 3mM β‐glycerophosphate, 50 μg/ml ascorbic acid and 10^‐9^ M dexamethasone. The isolated human adipose‐derived MSCs (AT‐MSCs) were cultured on the fabricated scaffolds with a cell density of 10^4^ cells/cm^2^ for biocompatibility tests such as cell attachment and viability assays and with a cell density of 3 × 10^4^ cells/cm^2^ for evaluating adipogenic and osteogenic differentiation as well as SEM analysis.

### 2.6 Plasmid construction, transfection and transduction

All plasmid construction, transfection and transduction processes were performed according to the previously reported protocol.^28^ MiR‐181a/b‐1 gene loaded Plenty‐III‐miR‐GFP plasmid (miR‐181a/b‐1 plasmid) for viral packaging was purchased from Shanghai Gene Pharma Co., Ltd. Biomaterials Science. The vector was transformed into Escherichia coli Stb14 cells, and then, plasmids were filtered via a plasmid extraction kit (iNtRON). After that, human embryonic kidney‐293 (HEK‐293) cells were thawed in culture Petri dishes (SPL, Korea) containing DMEM‐low glucose and 10% FBS. When the confluence of the HEK‐293T cells touched 70%‐80%, the miR‐181a/b‐1 plasmid was transfected into the cells. In 24, 36 and 48 hours after transfection, the medium was renewed and collected mediums were centrifuged at 1200 RPM for 5 minutes and, finally, ultra‐centrifuging was performed at 25 000 RPM for 2.5 hours for virus concentrating. Previously isolated and characterized AT‐MSCs were loaded into a 50‐ml falcon tube under FBS‐free DMEM supplemented with 8 µl of 2 mg/ml polybrene (Sigma‐Aldrich), and then, concentrated viruses were added to the AT‐MSCs with gentle pipetting. At the end of this process, FBS (10%) was also added to the cells and then aliquoted into several cell culture plates and incubated at 37°C with 5% CO_2_. The medium was exchanged every 12 hours during two days, and then, puromycin (Sigma‐Aldrich) was also added to the medium to screening only transduced AT‐MSCs.

### 2.7 Viability assay

The toxicity and biocompatibility of the fabricated scaffolds (PLGA and PLGA‐miR) compared to the tissue culture polystyrene (TCPS) were evaluated using the MTT test. MiR‐transduced AT‐MSCs cultured on the TCPS were considered as another group. Cultured scaffolds and TCPS were incubated at 37°C with 5% CO_2_ and on days 1, 5, 10 and 14 after cell seeding, MTT solution (5 mg/ml) was added to the cells and after 4 hours, supernatants were removed, and then, DMSO was added as a solvent of the formed Formazan. Finally, the solutions were collected and the optical density (OD) was measured at 570 nm using a microplate reader. Each group consisted of 3 repetitions.

### 2.8 Alkaline phosphates activity

ALP activity of the osteogenic differentiated AT‐MSCs cultured on fabricated scaffolds (PLGA and PLGA‐miR) compared to the TCPS was evaluated on days 7 and 14 after differentiation induction. MiR‐transduced AT‐MSCs cultured on the TCPS were considered as another group. The cells were removed from the substrates (scaffolds and TCPS) and then treated by RIPA cell‐lysis buffer for 1 hours at 4°C. Then, samples were centrifuged at 15 000 RPM for 15 minutes at 4°C. The supernatant containing the total protein was collected, and then, ALP activity and total protein were assessed using related kits (Beyotime) by measuring the OD at 450 nm using a microplate reader. Each group consisted of 3 repetitions.

### 2.9 Calcium measurement

Calcium content of the osteogenic differentiated AT‐MSCs cultured on fabricated scaffolds (PLGA and PLGA‐miR) compared to the TCPS was evaluated on days 7 and 14 after differentiation induction. MiR‐transduced AT‐MSCs cultured on the TCPS were considered as another group. To assay calcium, differentiated cells were removed from the substrates (scaffolds and TCPS) and then treated by HCL (0.6 N, Merck) for calcium release. After that, the calcium content reagent of the Beyotime kit was added to the samples and the OD was measured at 570 nm using a microplate reader. Calcium quantities in different samples were described after standardizing against the calcium serial dilution standard curve. Each group consisted of 3 repetitions.

### 2.10 Bone‐related gene expression

Runt‐related transcription factor 2 (Runx2), collagen type‐I (col‐I), osteonectin (OSN) and osteocalcin (OSC) gene expression was investigated in the differentiated cells cultured on the different groups (AT‐MSCs cultured on PLGA, PLGA‐miR and TCPS; miR‐transduced AT‐MSCs cultured on the TCPS) using real‐time RT‐PCR. On days 7 and 14 after differentiation induction, the cells were removed from the substrates and Qiazol (Qiagen) was added for extracting the total RNA. After that, a certain concentration of the extracted RNA was applied to synthesize complementary DNA (cDNA) using the Fermentas kit (Burlington, Ontario, Canada). By combining certain values of synthesized cDNA (5 μg/ml), injectable water, gene primer and SYBR Green Master Mix (Takara), a real‐time RT‐PCR process was performed by ABI StepOne‐plus instrument (Applied Biosystems). All primer sequences used in this study are mentioned in Table 1, while β‐2 M was considered as an internal control gene. Each group consisted of 3 repetitions.

**TABLE 1:** List of primers used in this study

Gene	Primer sequences	Size (bp)
β‐2‐Microglobulin (β 2 M)	TGGAAAGAAGATACCAAATATCGA	201
	GATGATTCAGAGCTCCATAGAGCT	
Runx2	GCCTTCAAGGTGGTAGCCC	67
	CGTTACCCGCCATGACAGTA	
Osteonectin (OSN)	AGGTATCTGTGGGAGCTAATC	224
	ATTGCTGCACACCTTCTC	
Osteocalcin (OSC)	GCAAAGGTGCAGCCTTTGTG	86
	GGCTCCCAGCCATTGATACAG	
Collagen I (col‐I)	TGGAGCAAGAGGCGAGAG	121
	CACCAGCATCACCCTTAGC	

### 2.11 Immunocytochemistry

The osteopontin protein as an important late bone‐related marker was evaluated in differentiated cells cultured on different groups (AT‐MSCs cultured on PLGA, PLGA‐miR and TCPS; miR‐transduced AT‐MSCs cultured on the TCPS) at the end of the study. The cells were fixed by treatment with paraformaldehyde (4%, Sigma‐Aldrich) at 4°C for 45 minutes. The samples were then blocked using the BSA solution (3%) for 1 hour at 4°C. The blocked samples were washed with PBS, and then, polyclonal rabbit anti‐human osteopontin (Alexis Biochemicals) was added and overnight incubated at 4‐8°C. The supernatants were removed and then washed again by PBS containing 0.05% Tween, and then, Alexa Fluor^®^ 488–conjugated goat anti‐rabbit IgG (Molecular Probes) was added to the cells as a secondary antibody for 1 hour. Before imaging with a fluorescence microscope, the samples were incubated by 4′, 6‐diamidino‐2‐phenylindole (DAPI) for 0.5 minutes for nuclear staining. Each group consisted of 3 repetitions.

### 2.12 Statistical analysis

REST2009 software was used for analysing the data acquired by real‐time RT‐PCR. The data were analysed using one‐way ANOVA and Tukey's post hoc multiple comparison test to evaluate the significance of the differences between groups (GraphPad Prism Software, version 9). All experiments were repeated three times. Values were expressed as mean ±standard deviation (SD). The *p‐*values less than 0.05 were declared as significantly significant.

## 3 RESULTS

### 3.1 Characterization of isolated Stem cells

**FIGURE 1:**
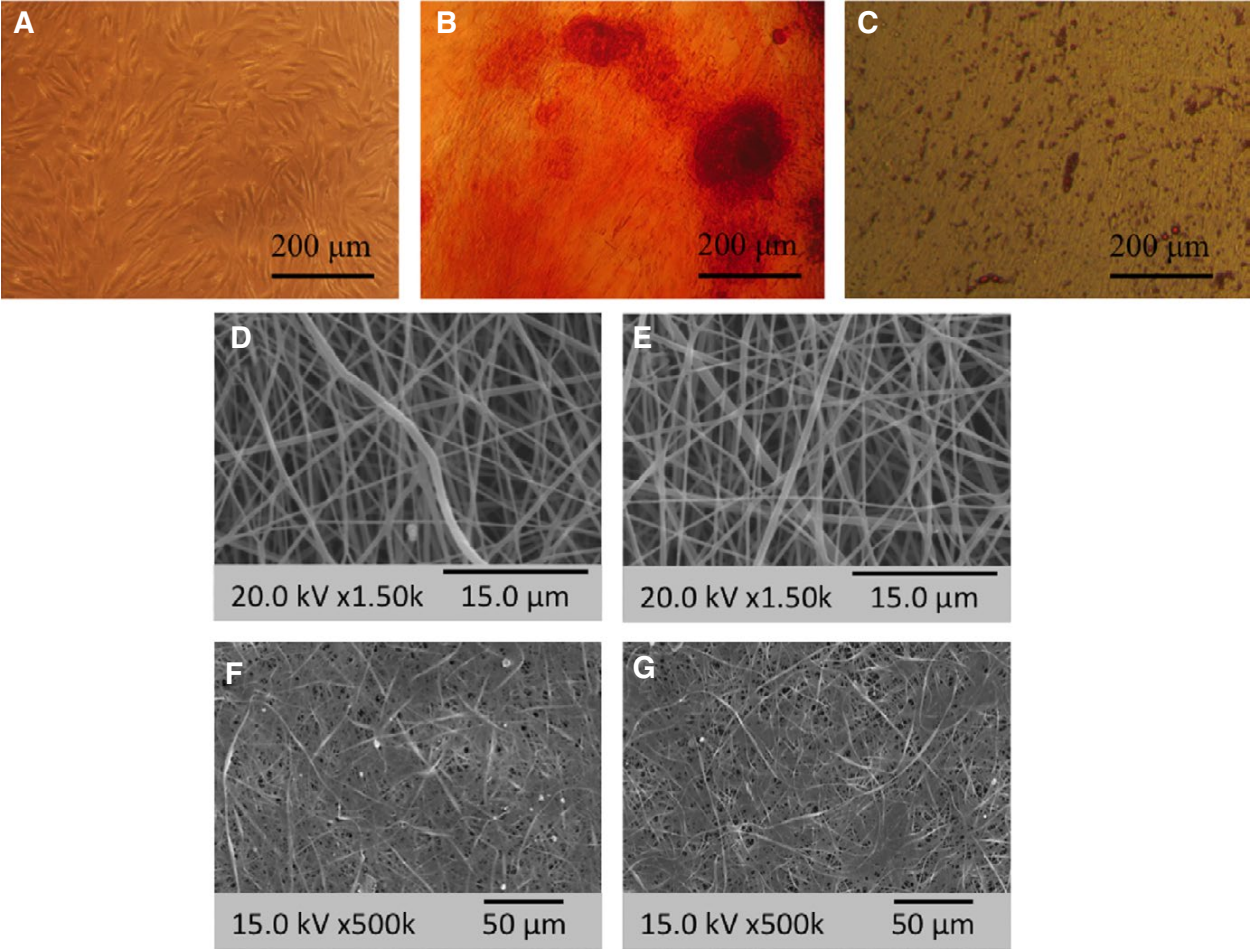
Human adipose‐derived mesenchymal stem cell (AT‐MSC) photographs at passage two while cultured under basal medium (A), while cultured under osteogenic medium for two weeks and then stained by Alizarin Red (B) and while cultured under adipogenic medium for two weeks and then stained by oil red (C). SEM photographs of PLGA nanofibres, before (D) and after cell seeding (F), and PLGA‐miR‐181a/b‐1 nanofibres before (E) and after cell seeding (G)

The isolated AT‐MSCs showed fibroblast‐like and spindle morphology (Figure 1A), as observed using light‐inverted microscopy. To evaluate the differentiation potential of the isolated AT‐MSCs, the cells were cultured under osteogenic and adipogenic differentiation medium and stained with Alizarin Red (Figure 1B) and oil red (Figure 1C) after two weeks to detect deposited calcium due to osteogenesis and oil vesicles due to adipogenesis, respectively. Thus, the results confirmed the differentiation of the isolated AT‐MSCs.

### 3.2 Characterization of Scaffolds

#### 3.2.1 Morphological awareness

Fabricated scaffolds were also characterized morphologically using SEM, and the results demonstrated that PLGA (Figure 1D) and PLGA‐miR (Figure 1E) were nanofibrous (823 ± 420 nm), bead‐free and smooth, and also incorporating miR does not have any significant impact on the scaffold morphology.

#### 3.2.2 Toxicity assessments

**FIGURE 2:**
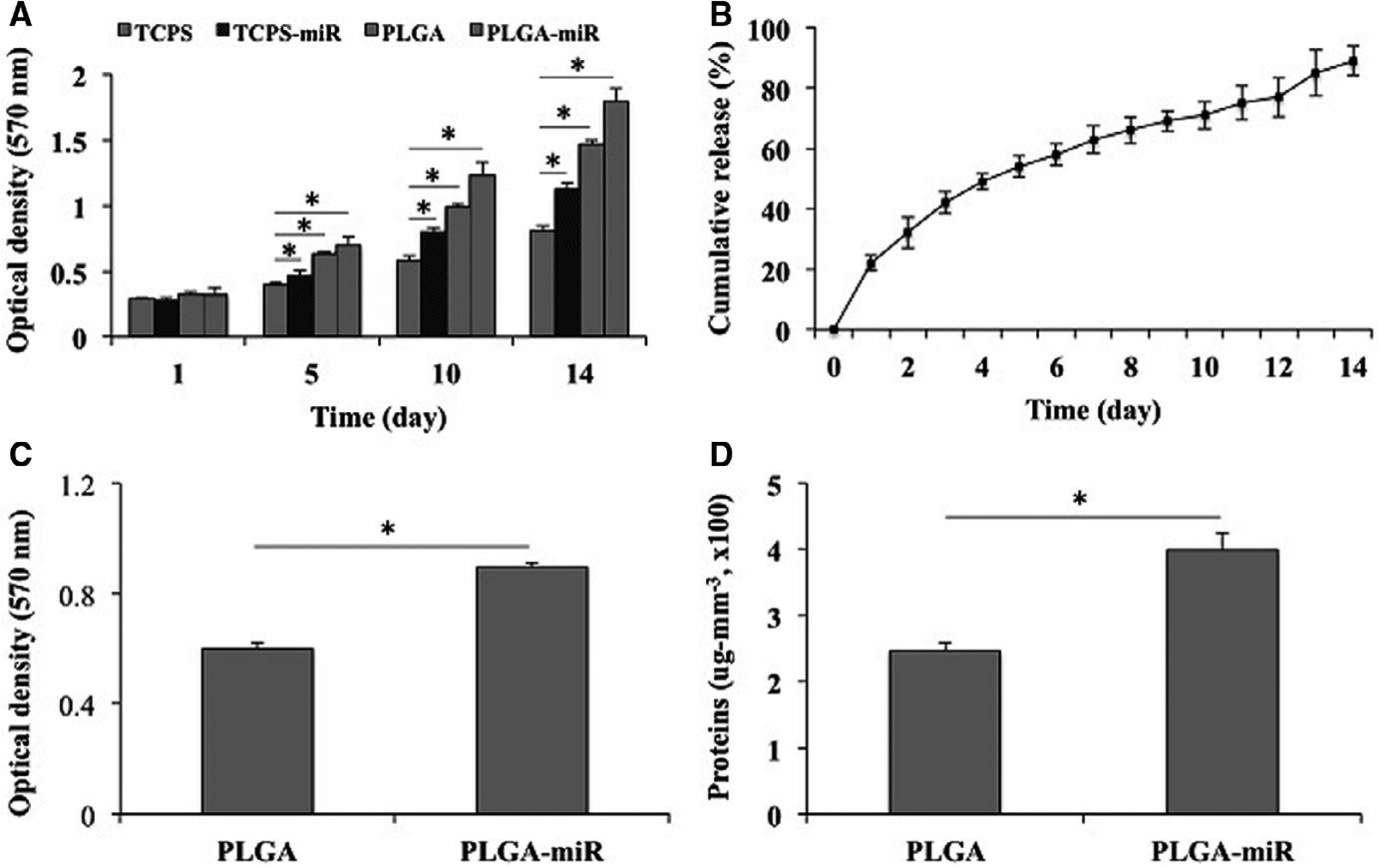
Viability evaluation of the human adipose‐derived mesenchymal stem cells (AT‐MSCs) when grown on the TCPS, PLGA and PLGA‐miR‐181a/b‐1 (PLGA‐miR), and miR‐transduced AT‐MSCs when grown on TCPS (A). Incorporated miR‐181a/b‐1 cumulative release profile from the PLGA nanofibres, during 14 days (B). Cell attachment (C) and protein adsorption (D) assays of the PLGA and PLGA‐miR‐181a/b‐1 (PLGA‐miR) nanofibrous scaffolds. The significant differences between groups are indicated with star sign (*P* < .05)

Several assays were performed to confirm the biocompatibility and non‐toxicity of the fabricated scaffolds. The AT‐MSCs were cultured on the PLGA (Figure 1F) and PLGA‐miR (Figure 1G) under basal medium and then visualized by SEM after two weeks. The results demonstrated that stem cells were grown, proliferated and expanded on the scaffolds. MTT assay was also performed to evaluate the viability of cells during 14 days (Figure 2A). The results revealed that except for the first day, in which there was no significant difference between the various groups, in other days, the highest survival rate was seen in the scaffold group containing miR. The survival rate of the cells cultured on the empty scaffold was also significantly higher than TCPS‐miR and TCPS groups. However, the survival rate detected in the TCPS‐miR group was also significantly higher than the TCPS group.

#### 3.2.3 Release profile assessment

Evaluating the release profile revealed that miR‐181a/b‐1 released from the nanofibres properly and continuously during the period of study, although on the first day a burst release of around 25% was detected (Figure 2B). For more biological behaviour evaluation of the fabricated scaffolds, cell attachment (Figure 2C) and protein adsorption (Figure 2D) assays were also performed. The results revealed that, in both assays, PLGA‐miR had a significantly higher value than empty PLGA.

### 3.3 Osteoinductive activity of scaffolds

#### 3.3.1 Calcium mineralization

**FIGURE 3:**
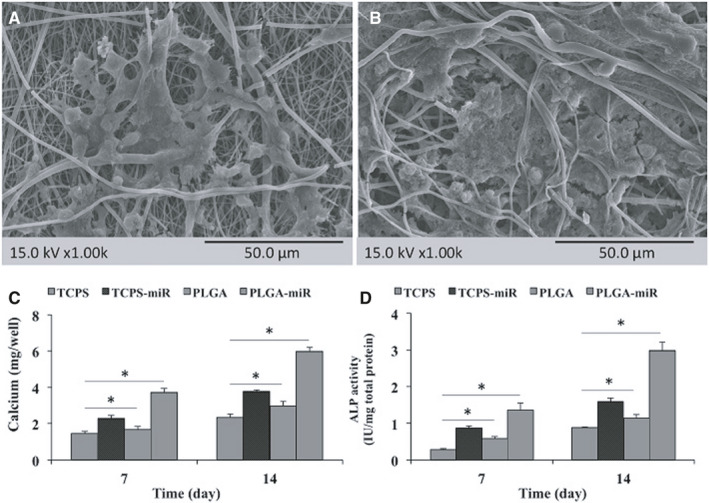
Deposited calcium depicted by SEM imaging when human adipose‐derived mesenchymal stem cells (AT‐MSCs) grown on the PLGA (A) and PLGA‐miR‐181a/b‐1 (PLGA‐miR) (B) nanofibrous scaffolds at the end of study. Calcium content (C) and ALP activity (D) of the AT‐MSCs when grown on the TCPS, PLGA and PLGA‐miR‐181a/b‐1 (PLGA‐miR), and miR‐transduced AT‐MSCs when grown on TCPS. The significant differences between groups are indicated with star sign (*P* < .05)

The SEM imaging performed at the end of the study verified calcium mineralization during osteogenesis of AT‐MSCs cultured on PLGA (Figure 3A) and PLGA‐miR (Figure 3B) nanofibrous scaffolds.

#### 3.3.2 Calcium content and ALP activity

The calcium content of the AT‐MSCs cultured on PLGA and PLGA‐miR nanofibrous scaffolds was also quantified, and the results revealed that the highest amount of calcium was detected in the PLGA‐miR group (Figure 3C). The calcium amount detected in the TCPS‐miR group was also increased significantly higher than the other two groups (TCPS and PLGA). Also, the calcium content of the PLGA group was significantly higher than the TCPS group.

ALP activity as an important osteogenic marker was investigated in the AT‐MSCs cultured on different substrates (Figure 3D). The results were similar to those obtained from the calcium content assay that showed the highest ALP activity in the PLGA‐miR group. In addition, ALP activity in the miR‐transduced AT‐MSCs cultured on TCPS was also significantly higher than the AT‐MSCs cultured on PLGA and TCPS.

#### 3.3.3 Expression of osteogenic‐related genes

**FIGURE 4:**
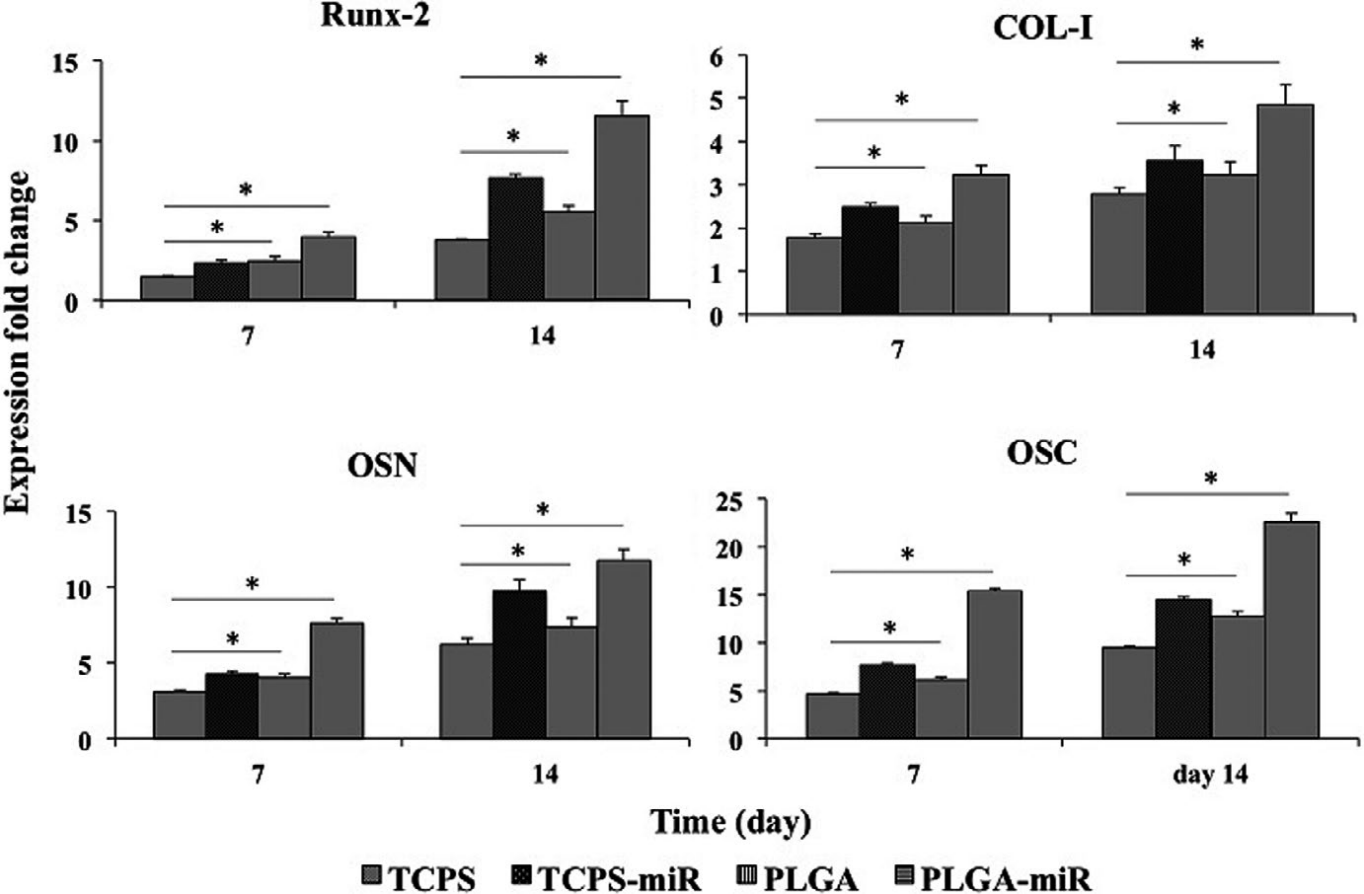
Bone‐related gene expression of the human adipose‐derived mesenchymal stem cells (AT‐MSCs) when grown on the TCPS, PLGA and PLGA‐miR‐181a/b‐1 (PLGA‐miR), and miR‐transduced AT‐MSCs when grown on TCPS. The significant differences between groups are indicated with star sign (*P* < .05)

The expression of osteogenic‐related genes was investigated in the cells cultured on different substrates (Figure 4). The results revealed that the highest expression level of Runx‐2 and OSN on days 7 and 14 was detected in the PLGA‐miR group. These gene expression levels in the TCPS‐miR and PLGA groups were not significantly different but were higher than the TCPS group on day 7. Besides, on day 14, the expression levels of Runx‐2 and OSN in the miR‐transduced AT‐MSCs cultured on the TCPS were significantly higher than the AT‐MSCs cultured on the PLGA and TCPS groups. Col‐I and OSC expression levels followed a similar pattern; the highest expression level of these genes was detected in the PLGA‐miR group on both days. Besides, these genes were also expressed in miR‐transduced AT‐MSCs cultured on the TCPS significantly higher than those in AT‐MSCs cultured on PLGA and TCPS on both days.

#### 3.3.4 Osteopontin protein expression

**FIGURE 5:**
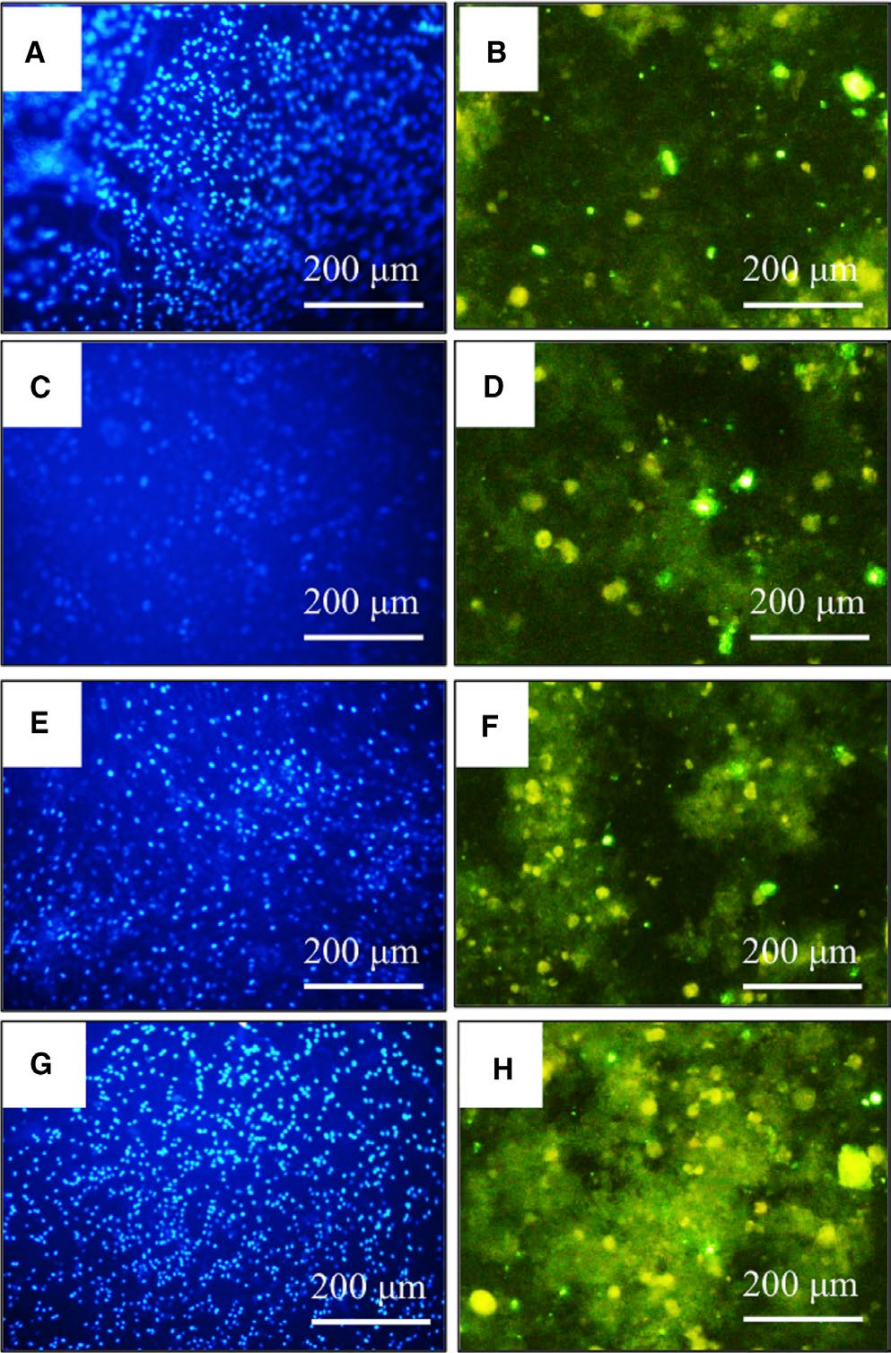
Nuclear staining (by DAPI) and osteopontin protein expression of the human adipose‐derived mesenchymal stem cells (AT‐MSCs) when grown on the TCPS (A and B), PLGA (E and F) and PLGA‐miR‐181a/b‐1 (PLGA‐miR) (G and H), and miR‐transduced AT‐MSCs when grown on TCPS (C and D), respectively

Osteopontin protein expression was also evaluated as an important late osteogenic marker at the end of the study (Figure 5). ICC results revealed that osteopontin was expressed significantly in the AT‐MSCs cultured on the PLGA‐miR nanofibrous scaffold (Figure 5H). However, the expression of osteopontin in the other groups, including AT‐MSCs cultured on TCPS (Figure 5B), and PLGA (Figure 5F), and miR‐transduced AT‐MSCs cultured on the TCPS (Figure 5D), was not significantly different.

## 4 DISCUSSION

Currently, repairing and replacing damaged tissues is the interest of researchers in the life sciences, medicine, and chemical and tissue engineering. Bone tissue is one of the tissues that usually need the most repair and transplantation due to its involvement in various types of injuries.^29^, ^30^ Although most bone injuries are healed spontaneously, conditions such as severe accidents, falls from heights, congenital cleft palate, periodontal disorders and residual lesions caused by bone cancer or various types of cancer require more precise treatment.^31^ Despite the current common treatments, there are many untreated defects and problems related to bone injuries in many people worldwide. Due to population growth, increasing average age of different communities, environmental pollution, injuries caused by war, stresses caused by machine life, etc, the number of patients who need bone repair and treatment is increasing.^32^, ^33^

Today, tissue engineering is the most important strategy for scientists in the production of tissue and prosthesis to repair or replace damaged tissues.^34^, ^35^ For bone tissue engineering, synthetic polymers are preferred, because of their superior mechanical properties to natural polymers.^36^ In addition to the structural similarity to the bone ECM, scaffolds can also induce signal pathways directly by storage and release of active biomolecules in situ.^3^

In the present study, miR‐181a/b‐1 was selected as an active biomolecule for incorporating in the nanofibrous PLGA scaffolds. Electrospinning was used as an easy, simple and accessible method for fabricating scaffolds.^37^ Characterization of the fabricated scaffolds demonstrated that incorporation of the miR‐181a/b‐1 does not have any significant effect on the size and diameter of the nanofibres. It was previously reported that there was no significant change in the structure and size of PLGA nanofibres when miR‐2861 was incorporated in the fibres.^15^ On the other hand, the biocompatibility of the nanofibres was significantly increased, when incorporating with miR‐181a/b‐1. The amounts of cell attachment and protein adsorption were also increased in miR‐incorporated nanofibres compared to the empty nanofibres. In addition, MTT assay was revealed that the viability of the AT‐MSCs was significantly increased while growing on the PLGA‐miR nanofibres compared to the cells cultured on the PLGA and TCPS. The release of miR‐181a/b‐1 from the nanofibres continued steadily for two weeks, which was predictable due to the polymer properties. In line with our results, Tahmasebi et al demonstrated that the viability and proliferation rate of the human iPSCs were increased while cultured on the miRNA‐incorporated polycaprolactone (PCL) nanofibres after a week compared to the empty PCL.^14^ In addition, the positive impact of miR‐181a on the proliferation rate of the AGS, SGC‐7901, 293 and Jurkat cells was also reported previously.^38^, ^39^

Furthermore, to evaluate osteoinductivity of the fabricated nanofibres, ALP activity, calcium content, and bone‐related gene and protein expression assays were performed for AT‐MSCs cultured on the TCPS, PLGA and PLGA‐miR groups, while miR‐transduced AT‐MSCs cultured on the TCPS was considered as another group. The obtained results revealed that miR‐181a/b‐1 has great osteoinductive potential and this potential is significantly increased while incorporating in the nanofibrous structure of PLGA. ALP activity and calcium measures of the miR‐transduced AT‐MSCs cultured on the TCPS were significantly increased in comparison with those AT‐MSCs cultured on TCPS. But, these increases in ALP activity and calcium measures were significantly enhanced when AT‐MSCs cultured on the PLGA‐miR nanofibres, indicating the synergistic effect of miR‐181a/b‐1 and PLGA nanofibre. It was demonstrated that reconstruction of the rat calvarial defect was improved significantly when implanted by PLGA nanofibres.^40^ It was recently revealed that miR‐181a/b‐1 can stimulate osteogenesis by targeting the PTEN/PI3K/AKT signal pathway through modulating mTORC1.^41^ Altogether, the results of the present study indicate that miR‐181a/b‐1 combining with PLGA nanofibres has great potential to use as osteoinductive agents in patients suffering from osteoporosis or other bone lesions. The main challenge of this project was to encapsulate an appropriate amount of microRNA in nanofibres. It is suggested that the electrospinning time be increased to produce a thicker scaffold and that further studies be performed in animal models to confirm scaffolds effect on bone repair.

## 5 CONCLUSION

According to the results, it can be concluded that incorporating the miR‐181a/b‐1 not only has any significant effect on the PLGA nanofibres structure but also has a significant positive impact on the biocompatibility of the PLGA nanofibres. In addition, the osteogenic differentiation capacity of the human AT‐MSCs was increased while transduced by miR‐181a/b‐1, and this capacity was also synergistically increased while miR‐181a/b‐1 incorporated into PLGA nanofibres. Taking to gather, the results demonstrated that the combination of miR‐181a/b‐1 and PLGA nanofibrous scaffold could be considered as a promising potential candidate for bone tissue engineering applications.

## CONFLICT OF INTEREST

The authors confirm that there are no conflicts of interest.

## AUTHOR CONTRIBUTION

**Peiyi Qi**: Conceptualization (equal); Funding acquisition (lead); Supervision (lead); Validation (lead). **Yali Niu**: Data curation (equal); Investigation (equal); Writing‐original draft (equal). **Bin Wang**: Data curation (equal); Investigation (equal); Writing‐review & editing (equal).

## Data Availability

The data based on the results of the current study were obtained and are accessible from the corresponding authors upon reasonable request.
